# Preoperative Antithrombotic Exposure and Postoperative Outcomes Following Nontraumatic Subdural Hematoma Evacuation: A Multicenter Electronic Health Record Analysis with Single-Center Context

**DOI:** 10.3390/brainsci16101084

**Published:** 2026-10-09

**Authors:** Noah B. Drewes, Lara Koutah, Rishi Jain, Hannah E. Johnson, Jack Carnduff, Khizar R. Nandoliya, Kayla Chin, Ava Hoeft, Savannah Kidd, Alexander C. Kowalski, Kevin Lu, Hannah Roberts, Yixuan Ma, Guadalupe Ruiz, Kyle Struck, Jessica Sheelam, Dylan J. Thompson, Faris Shaikh, Krish Patel, Kristin R. Delfino, Bruce M. Frankel

**Affiliations:** 1 Department of Surgery, Division of Neurosurgery, Southern Illinois University School of Medicine, 801 N. Rutledge St., PO Box 19620, Springfield, IL 62702, USAjcarnduff76@siumed.edu (J.C.); akowalski98@siumed.edu (A.C.K.); gruiz29@siumed.edu (G.R.); fshaikh86@siumed.edu (F.S.); mfrankel62@siumed.edu (B.M.F.); 2 Department of Neurological Surgery, Feinberg School of Medicine, Northwestern University, 676 N. St. Clair St., Suite 2210, Chicago, IL 60611, USA; larakoutah2025@u.northwestern.edu (L.K.);; 3 Department of Neurosurgery, John Sealy School of Medicine, The University of Texas Medical Branch, 301 University Blvd., Galveston, TX 77555, USA; 4 Center for Clinical Research, Southern Illinois University School of Medicine, 201 E. Madison St., Springfield, IL 62702, USA; kdelfino84@siumed.edu

**Keywords:** subdural hematoma, antithrombotic therapy, anticoagulants, antiplatelets, reoperation

## Abstract

**Highlights:**

**What are the main findings?**

- Preoperative antithrombotic use was associated with a higher likelihood of repeat SDH surgery in the multicenter EHR cohort, but this pattern was not reproduced in the single-center cohort.
- Mortality and thrombotic outcomes were not associated with antithrombotic exposure after accounting for clinical characteristics.

**What are the implications of the main findings?**

- Antithrombotic exposure may be a marker of a medically complex SDH population as opposed to a sole predictor of poor postoperative outcomes.
- Postoperative surveillance and antithrombotic management should be individualized according to each patient’s hemorrhagic and thrombotic risk.

**Abstract:**

**Background/Objectives**: Preoperative antithrombotic therapy is common among patients undergoing subdural hematoma (SDH) evacuation. However, the association with postoperative outcomes remains uncertain. We evaluated this potential association using a multicenter electronic health record cohort and a single-center cohort. **Methods**: We performed a retrospective cohort study using TriNetX, a multicenter electronic health record (EHR) database, and a single-center cohort. TriNetX was used to identify adults undergoing evacuation of nontraumatic subdural hematoma between 2005 and 2025. The exposed cohort was defined as documented oral anticoagulant or antiplatelet exposure within 7 days before surgery, after which patients were propensity-score matched 1:1. The single-center cohort included surgically treated subdural hematoma patients with clinical, radiographic, laboratory, and operative data. Postoperative outcomes included 30-day mortality, short-term thrombotic complications, and 90-day repeat subdural hematoma surgery. **Results**: In TriNetX, 15,651 eligible patients were identified. Matched cohorts included 1455 exposed and 1455 unexposed patients. Preoperative antithrombotic exposure was associated with higher 90-day repeat surgery (9.7% vs. 6.5%; hazard ratio [HR] 1.52; *p* = 0.001), whereas 30-day mortality and 14-day thrombotic complications did not differ significantly. The single-center cohort included 164 surgically treated patients, including 103 exposed and 61 unexposed patients. The reoperation association was not reproduced. However, exposed patients had higher unadjusted 30-day and overall mortality that attenuated after multivariable Cox adjustment. **Conclusions**: Preoperative antithrombotic exposure was associated with repeat surgery in the TriNetX cohort but not in the single-center cohort. Together, these findings suggest that antithrombotic exposure may identify a medically complex surgical SDH population requiring individualized postoperative surveillance.

## 1. Introduction

Subdural hematoma (SDH) is among the most frequently encountered cranial neurosurgical conditions, and heterogeneity exists in how this pathology presents, ranging from headaches to coma and death [1]. Head trauma is a common cause, though the initial injury may be minor or forgotten. Additionally, some hematomas may occur without an obvious traumatic history [1,2]. Older individuals are especially impacted by nontraumatic SDH, which often occurs in the setting of cerebral atrophy, comorbidities, and antithrombotic exposure [2,3]. Other notable predisposing factors include coagulopathy or excessive alcohol use [1,2]. The burden of SDH is rising, with reported chronic SDH incidence ranging from about 2 to 58 cases per 100,000 person-years, with the elderly population at the highest risk [4,5,6]. As the population ages and antiplatelet and anticoagulant use becomes more common, postoperative SDH management increasingly requires balancing recurrent hemorrhage risk against preventing thrombotic complications [7,8].

Management of symptomatic SDH includes observation or surgical evacuation through twist-drill, burr-hole, or craniotomy, which largely depends on the clinical presentation and hematoma characteristics [1,7]. Middle meningeal artery embolization is emerging as an adjunctive or alternative strategy in some chronic SDH populations [9,10]. Despite aggressive treatment methods, recurrence remains a major limitation, with pooled recurrence after burr-hole drainage estimated at around 13% [11]. 

Following surgical management of SDH, postoperative prognosis is influenced by multiple patient-related, radiographic, and operative variables. In chronic SDH studies, older age and poorer functional status have been associated with worse outcomes [6,12]. Other comorbidities such as heart failure and advanced renal disease have also been associated with a deterioration in living activities [13]. Markers of frailty, such as a dependence in daily activities or a greater comorbidity burden, have also been associated with higher rates of mortality following chronic SDH [14,15]. Hematoma thickness, midline shift, internal architecture, and extent of evacuation have been associated with recurrence or reoperation, while postoperative drain placement can reduce recurrence [16,17,18]. Overall, prior studies necessitate considering clinical status, hematoma characteristics, and surgical management when stratifying risks for postoperative SDH outcomes.

Indeed, antithrombotic therapy is associated with SDH development and may contribute to rising SDH incidence [19]. However, whether preoperative antiplatelet or anticoagulant exposure is associated with worse postoperative outcomes remains unclear, as prior studies are often single-center, variably define recurrence, and do not fully address confounding by comorbidity [20,21,22]. Importantly, patients prescribed these medications often have underlying vascular or thromboembolic disease, and their higher comorbidity burden may influence prognosis [20,21]. 

Therefore, we evaluated the association between preoperative antithrombotic exposure and postoperative outcomes following surgical evacuation of SDH using TriNetX and a single-center cohort. The TriNetX US Collaborative Network was used to estimate associations between preoperative anticoagulant or antiplatelet (AC/AP) exposure and postoperative mortality, thrombotic complications, and repeat surgery in a large propensity-score-matched TriNetX cohort. A separately sourced single-center cohort was used to provide chart-reviewed AC/AP medication histories, as well as clinical, radiographic, and laboratory data that are unavailable in TriNetX.

## 2. Materials and Methods

### 2.1. Study Design

This retrospective cohort study used both the TriNetX multicenter electronic health record (EHR) database (TriNetX, LLC, Cambridge, MA, USA) and a single-center cohort and was reported in accordance with the Strengthening the Reporting of Observational Studies in Epidemiology (STROBE) guidelines. The TriNetX US Collaborative Network, a research platform combining deidentified health records from multiple organizations, was queried to perform this study. This multicenter network combines data from participating US healthcare organizations and enables analyses of deidentified and routinely collected EHR data within these organizations. These data include demographics, diagnoses, operations, prescriptions, and laboratory values. Within TriNetX, mutually exclusive exposed and unexposed groups were generated among patients undergoing operative evacuation of nontraumatic SDH. Patients were classified as exposed when they had documented use of at least one oral anticoagulant or antiplatelet medication within 7 days of surgical intervention. Unexposed patients had no recorded antithrombotic use during the same period. Exposure was defined by documented preoperative AC/AP therapy. In this study, diagnoses and outcomes were chosen based on terminology from the International Classification of Diseases, Tenth Revision (ICD-10), procedures from the Current Procedural Terminology (CPT), and medication terminology from RxNorm.

A single-center cohort of surgically treated SDH patients was analyzed to provide clinical, radiographic, and laboratory data not available in TriNetX. The TriNetX and single-center cohorts were analyzed independently and were not pooled.

The TriNetX analysis used deidentified data and was exempt from institutional review board review. The single-center cohort was approved by the local institutional review board (approval #24-464), and informed consent was waived.

### 2.2. Cohort Synthesis

For the TriNetX cohort, eligible patients were adults aged ≥18 years undergoing operative evacuation of SDH between 1 September 2005 and 1 September 2025. CPT codes, including twist-drill (61108), burr-hole evacuation (61154), or craniotomy (61312, 61314), were used to identify surgical evacuation. A diagnosis of nontraumatic SDH (ICD-10 I62.0x) within 0–7 days before the procedure was required. Nontraumatic SDH refers to the coded etiology available in TriNetX and does not specify hematoma chronicity or mechanism. Importantly, clinically derived chronicity was not available to stratify into groups for the present analysis. Patients were excluded if they were aged <18 years old, received thrombolytics within 7 days before or after the procedure, which included alteplase, tenecteplase, reteplase, streptokinase, urokinase, anistreplase, ancrod, drotrecogin alfa, or protein C, or underwent surgery outside the study period. A full list of the TriNetX codes for cohort inclusion is detailed in Table S1.

For the single-center analysis, we queried all patients who were evaluated for SDH at our institution between 2008 and 2024. We used a broad diagnosis-code search (ICD-10-CM I62 and ICD-9-CM 432.1 and 432.9) to identify potentially eligible SDH cases at our institution between 2008 and 2024. Each record was reviewed manually to determine study eligibility, including whether the SDH was nontraumatic based on the documented history. Patients were excluded if their age was <18 years old, as well as if they sustained a high-energy trauma, had clinically significant coexisting intracranial pathology such as an intraparenchymal or subarachnoid hemorrhage, had a non-convexity SDH, had insufficient medical records, or underwent conservative management. Therefore, the SDH selection was not identical to the code-based TriNetX definition. In the single-center cohort, hematoma chronicity could be abstracted rather than inferred from the term nontraumatic. The final included cohort consisted of 164 surgically managed SDH patients.

Demographics, comorbidities, Glasgow Coma Scale (GCS) score at presentation, INR, platelet count, SDH stage, laterality, maximum hematoma thickness, midline shift, and operative approach were all abstracted during chart review and confirmed by two independent reviewers. Outcome data collected included discharge discharge modified Rankin Scale (mRS) score, thrombotic events during the index hospitalization, reoperation, and mortality.

In both TriNetX and the single-center cohort, preoperative exposure to AC/AP therapy was defined as the presence of documented AC/AP use within 7 days before surgical intervention for SDH. Patients were then stratified as any exposure to AC/AP therapy versus no exposure. In a sensitivity analysis, exposed patients in either cohort were classified into mutually exclusive anticoagulant-only, antiplatelet-only, and dual anticoagulant/antiplatelet groups, and were all compared with unexposed patients to assess whether any associations were driven by a single drug class.

### 2.3. Exposures

In TriNetX, two cohorts were created among patients undergoing surgical management for SDH. A group for preoperative AC/AP exposure included patients with ≥1 prescription for oral warfarin, apixaban, rivaroxaban, dabigatran etexilate, edoxaban, aspirin, clopidogrel, prasugrel, ticagrelor, or ticlopidine within 7 days before the index SDH evacuation. The unexposed group included patients with no record of oral, parenteral, or intravenous antithrombotic agents within 7 days prior to surgery. In the single-center cohort, preoperative AC/AP exposure was defined as documented anticoagulant or antiplatelet use within 7 days before presentation or surgery. After the cohorts were synthesized, further classification into mutually exclusive AC-only, AP-only, and dual AC+AP groups, with dual therapy defined as exposure to at least one agent from each class, generated subgroups for sensitivity analyses.

### 2.4. Outcomes of Interest

For the TriNetX cohort, the outcomes queried included a repeat SDH surgery within 90 days, postoperative mortality within 30 days, and thrombotic complications within 14 days, which consisted of ischemic stroke, myocardial infarction, deep venous thrombosis, and pulmonary embolism. To evaluate repeat surgery in TriNetX, the same CPT codes used to define the index surgery were queried a second time.

In the single-center cohort, the primary outcomes of interest included 90-day reoperation, thrombotic events during the index hospitalization, and 30-day postoperative mortality. Secondary outcomes included the mRS at discharge and overall survival. The thrombotic events included were the same as those collected in TriNetX. Discharge mRS was dichotomized as good outcome, mRS 0–3, versus poor outcome, mRS 4–6.

The window for capturing thrombotic events differed between cohorts. Reliably restricting outcomes to the index hospitalization was not possible in TriNetX; therefore, outcomes were based on a fixed postoperative 14-day window, whereas single-center events were abstracted from charts during the index hospitalization, regardless of length of stay.

### 2.5. Statistical Analysis

AC/AP-exposed and unexposed patients were matched 1:1 using greedy nearest-neighbor propensity-score matching. The baseline covariates of interest were age, sex, race, ethnicity, and body mass index (BMI). Other covariates were also accounted for, which was largely based on common prescribing practices for antithrombotic therapies. These included atrial fibrillation or flutter, venous thromboembolism, pulmonary embolism, ischemic heart disease, peripheral vascular disease, cerebral infarction, transient ischemic attack, essential hypertension, type 2 diabetes mellitus, chronic kidney disease, heart failure, liver disease, alcohol-related disorders, mechanical heart valves, and nicotine dependence. For balance to be considered acceptable, a standardized mean difference (SMD) below 0.10 was required. Age was included in the model; however, modest residual imbalance remained after cohort matching. Because TriNetX does not allow for further improvement in balance, this was retained as residual imbalance and acknowledged as a source of potential confounding.

In TriNetX, 30-day mortality and 90-day repeat SDH surgery were analyzed as time-to-event outcomes using Kaplan–Meier curves with hazard ratios (HRs). Fourteen-day thrombotic complications were analyzed as binary outcomes rather than time-to-event endpoints, and risk ratios (RRs) were therefore calculated.

Similarly, in the single-center cohort, 30-day mortality and 90-day reoperation were assessed as time-to-event outcomes using Kaplan–Meier curves and hazard ratios. Thrombotic complications were analyzed as binary events during the index hospitalization and were summarized using risk ratios. For 30-day mortality and overall survival, both univariable and multivariable Cox proportional hazards models were conducted. The 30-day mortality multivariable Cox model included AC/AP exposure, age, tobacco status, hypertension, atrial fibrillation, and coronary artery disease. The overall survival model included these variables, in addition to discharge mRS. Continuous variables were compared using Wilcoxon rank-sum tests, and categorical variables using chi-square or Fisher exact tests. Statistical significance was defined as *p* < 0.05. Figures were generated using R (version 4.1.2; R Foundation for Statistical Computing, Vienna, Austria).

The same analyses used in the primary analysis were done for the sensitivity analyses comparing mutually exclusive AC-only, AP-only, and dual AC+AP groups with unexposed controls, with identical 1:1 propensity-score matching performed for each comparison in TriNetX. In the single-center cohort, sensitivity analyses compared the same mutually exclusive groups with unexposed patients. Baseline characteristics were compared using Kruskal–Wallis, Pearson chi-square, or Fisher–Freeman–Halton tests. Unadjusted Cox models were used to assess 90-day reoperation and 30-day mortality, and thrombotic events were analyzed using risk ratios.

Competing-risk analyses were additionally conducted that treated death within 90 days before reoperation as the competing event. In TriNetX, Aalen–Johansen estimates were obtained from the any-AC/AP and unexposed cohorts before matching. The analyses available in the network did not provide matched competing-risk regression. In the single-center cohort, follow-up ended at the first reoperation, death, or day 90. Any-AC/AP and unexposed cohorts were compared using Gray’s test and unadjusted Fine–Gray regression, which allowed for different censoring patterns between exposure groups. Deaths after reoperation were not classified as competing events.

## 3. Results

### 3.1. Study Population

**Figure 1:**
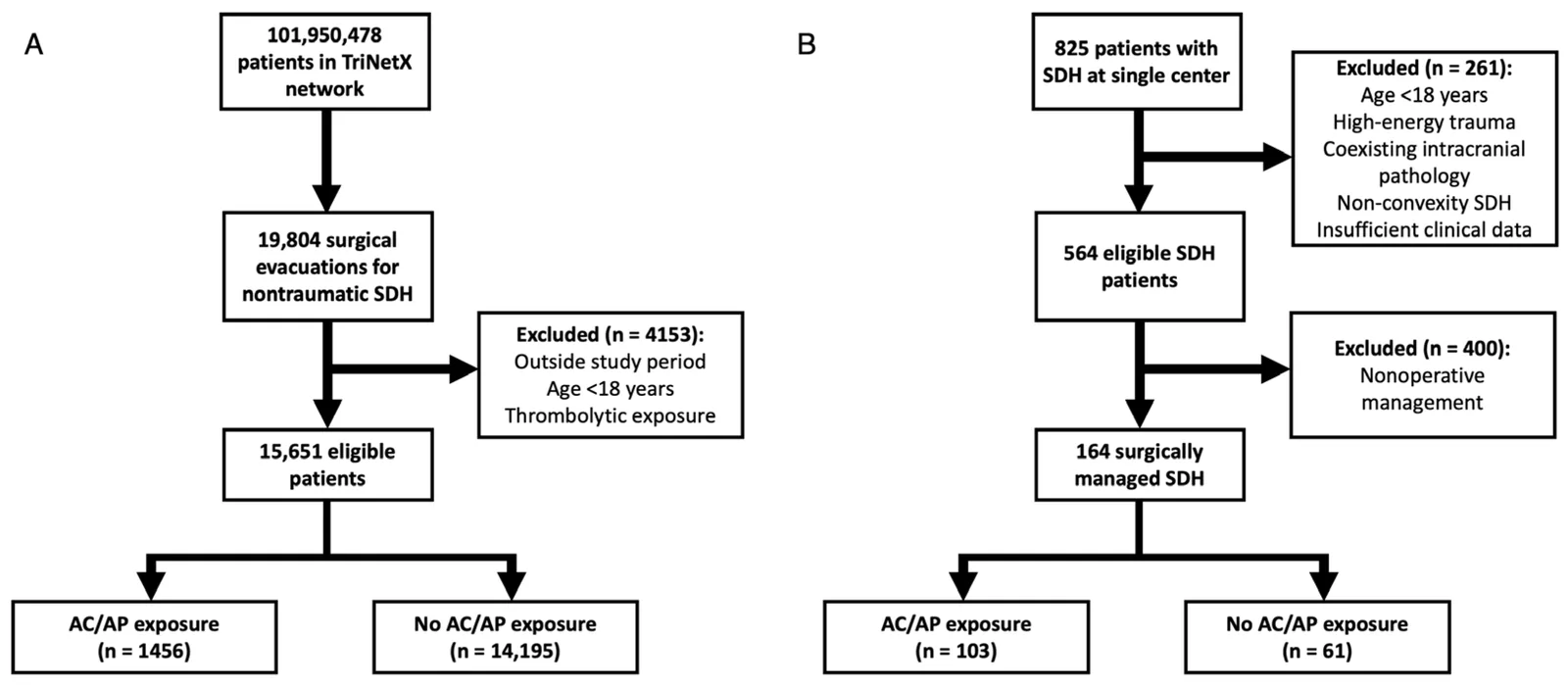
TriNetX and single-center cohort derivation. (**A**) TriNetX cohort derivation using TriNetX to identify adults undergoing operative evacuation of nontraumatic subdural hematoma, stratified by documented preoperative anticoagulant or antiplatelet exposure before propensity-score matching. (**B**) Single-center cohort derivation of surgically treated subdural hematoma patients stratified by preoperative anticoagulant or antiplatelet exposure. Abbreviations: AC/AP, anticoagulant/antiplatelet; SDH, subdural hematoma.

After exclusion criteria were applied, 15,651 total patients were eligible that had undergone surgical evacuation of nontraumatic SDH in the TriNetX US Network. After applying 1:1 propensity-score matching, there were 1455 patients included in each cohort: those with exposure to preoperative AC/AP therapy and those without exposure (Figure 1A). Most of the baseline demographic and clinical variables demonstrated adequate balance, as standardized mean differences were <0.10. Age remained modestly imbalanced after matching (69.0 vs. 70.6 years; SMD = 0.14; Table 1).

The single-center cohort started with 825 SDH patients. Of these, 261 were excluded due to fulfilling at least one of the following criteria: age < 18 years, high-energy trauma, coexisting intracranial pathology, non-convexity SDH, or insufficient clinical information. An additional 400 were excluded for nonoperative management. The final study cohort consisted of 164 surgically treated SDH patients. Of these, 103 had preoperative AC/AP exposure, and 61 had none (Figure 1B). Clinical, radiographic, laboratory, operative, and functional characteristics for the single-center cohort are demonstrated in Table 2. Overall, patients with AC/AP exposure were older, had higher BMI, and were more likely to have hypertension, atrial fibrillation, and coronary artery disease. Additionally, they had higher INR and lower platelet counts at presentation. Radiographic measurements such as maximum hematoma thickness and midline shift were similar between groups. Craniotomy or craniectomy was the most common operative approach in both groups. Poor discharge mRS (4–6) was more frequent among AC/AP-exposed patients. Presenting symptoms were similar between groups (Table S2).

### 3.2. TriNetX Postoperative Outcomes

**Figure 2:**
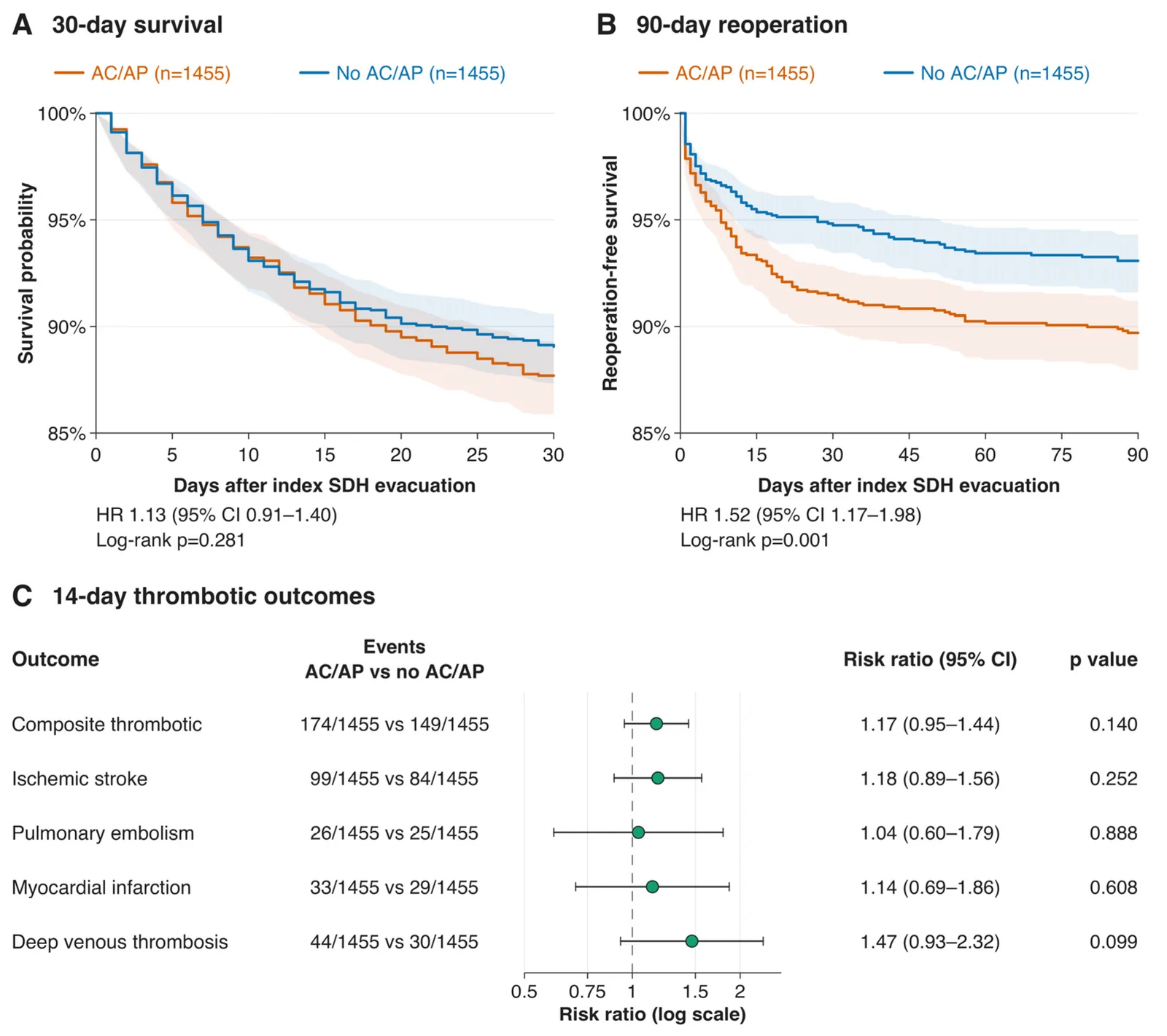
TriNetX postoperative outcomes after SDH evacuation. Kaplan–Meier and forest plot analyses from the propensity score-matched TriNetX cohort comparing patients with and without preoperative anticoagulant or antiplatelet exposure. (**A**) Thirty-day survival. (**B**) Freedom from 90-day repeat SDH surgery. (**C**) Fourteen-day thrombotic complications, including the composite endpoint and individual thrombotic event subtypes. Abbreviations: AC/AP, anticoagulant/antiplatelet; CI, confidence interval; HR, hazard ratio; SDH, subdural hematoma.

For those in the TriNetX analysis, we found that mortality occurred in 176 (12.1%) patients exposed to AC/AP and in 157 (10.8%) unexposed patients within 30 days after the index procedure (HR 1.13, 95% confidence interval [CI] 0.91–1.40; *p* = 0.281; Figure 2A, Table 3). Reoperation took place in 141 (9.7%) exposed patients, whereas 94 (6.5%) unexposed patients underwent a repeat surgery (HR 1.52, 95% CI 1.17–1.98; *p* = 0.001; Figure 2B, Table 3), showing an association between preoperative AC/AP exposure and reoperation within 90 days. The overall thrombotic complication rate at 14 days included 174 (12.0%) in the AC/AP group and 149 (10.2%) in the control group, which was not statistically significant (RR 1.17, 95% CI 0.95–1.44; *p* = 0.140; Figure 2C, Table 3). A breakdown of the individual thrombotic outcomes is located in Table S3.

### 3.3. Single-Center Postoperative Outcomes

**Figure 3:**
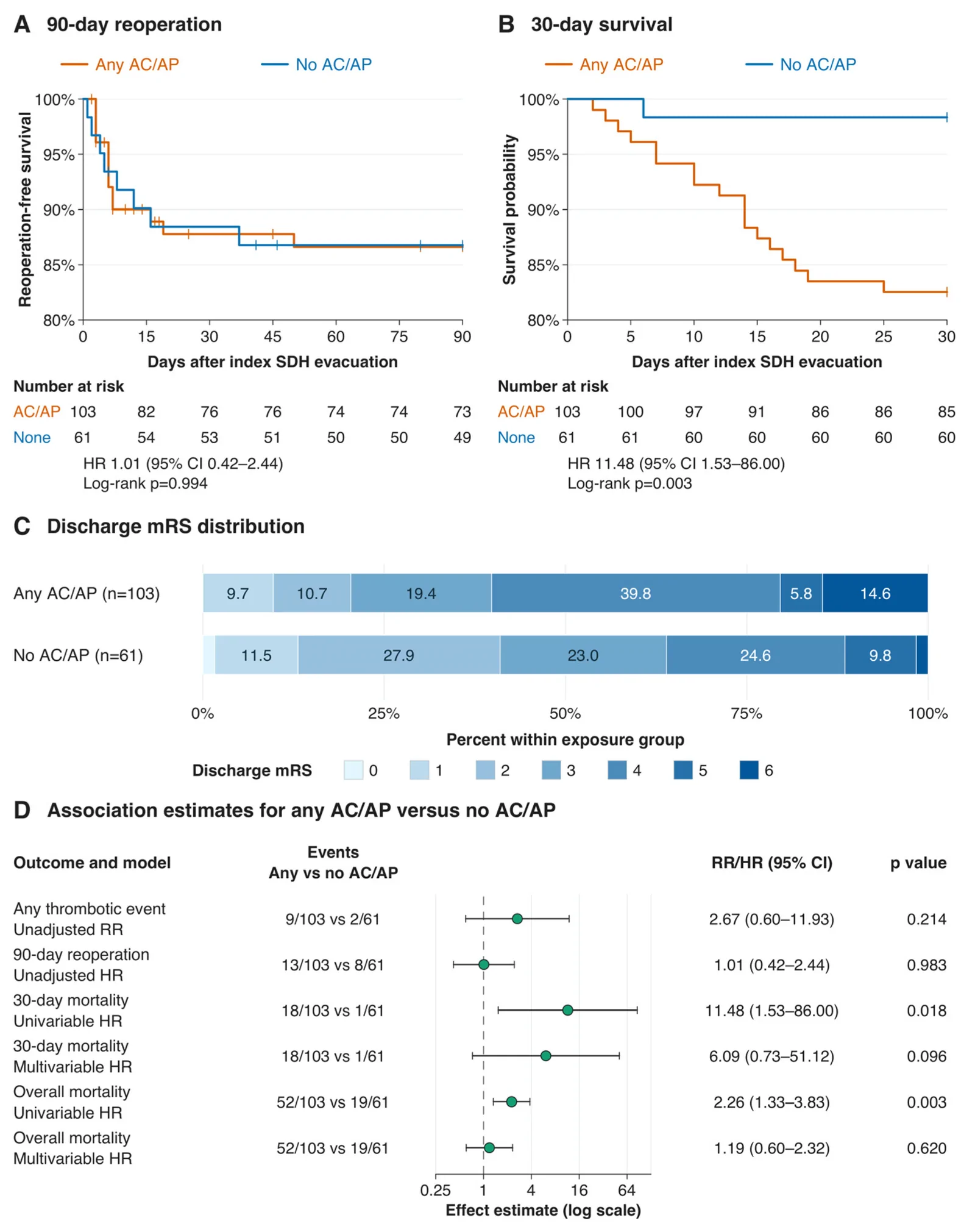
Single-center cohort outcomes and granular clinical context. Single-center analyses comparing surgically treated SDH patients with and without preoperative anticoagulant or antiplatelet exposure. (**A**) Freedom from 90-day reoperation. (**B**) Thirty-day postoperative survival. (**C**) Discharge modified Rankin Scale distribution by exposure status. (**D**) Exposure–outcome model estimates for thrombotic events, reoperation, 30-day postoperative mortality, and overall survival. Hazard ratios for mortality use death as the event. Abbreviations: AC/AP, anticoagulant/antiplatelet; CI, confidence interval; HR, hazard ratio; mRS, modified Rankin Scale; RR, risk ratio; SDH, subdural hematoma.

In the single-center analysis, reoperation within 90 days occurred in 13 (12.6%) exposed and 8 (13.1%) unexposed patients and did not differ by exposure status (HR 1.01, 95% CI 0.42–2.44; *p* = 0.983; Figure 3A, Table 3). Within this cohort, 30-day mortality differed by exposure status, occurring in 18 (17.5%) exposed patients and 1 (1.6%) unexposed patients (log-rank *p* = 0.003). Univariable Cox analysis showed a higher hazard of 30-day mortality among exposed patients (HR 11.48, 95% CI 1.53–86.00; *p* = 0.018). However, after multivariable Cox adjustment, this association attenuated (HR 6.09, 95% CI 0.73–51.12; *p* = 0.096; Figure 3B,D, Tables S4 and S5). Discharge mRS was worse among exposed patients. A poor discharge mRS occurred in 62 (60.2%) exposed and 22 (36.1%) unexposed (Figure 3C, Table 2). Thrombotic events occurred in 9 (8.7%) exposed patients and 2 (3.3%) unexposed (RR 2.67, 95% CI 0.60–11.93; *p* = 0.214; Figure 3D, Table S3). 

Overall survival differed in univariable analysis, with preoperative exposure showing a higher hazard of mortality with preoperative AC/AP exposure (HR 2.26, 95% CI 1.33–3.83; *p* = 0.003; Figure S1). This association was not significant after multivariable adjustment (HR 1.19, 95% CI 0.60–2.32; *p* = 0.620; Figure 3D, Tables S6 and S7).

The overall outcome patterns for both TriNetX and the single-center analysis are demonstrated in Table 3. The TriNetX cohort showed a significant association between preoperative AC/AP exposure and 90-day repeat SDH surgery. However, this was not observed in the single-center analysis. The single-center cohort displayed greater 30-day mortality that attenuated after multivariate adjustment. Both cohorts showed a numerically higher number of thrombotic complications in the preoperative exposure groups, but statistical significance was not reached.

### 3.4. Sensitivity Analyses

In the drug-class sensitivity analysis, the single-center groups were composed of 61 unexposed, 20 AC-only, 63 AP-only, and 20 dual-exposure patients (Table S8). There were no class-specific reoperation or thrombotic comparisons that were statistically significant. Unadjusted 30-day mortality HRs were elevated for AP only (HR 9.18, 95% CI 1.16–72.47; *p* = 0.035) and dual exposure (HR 20.57, 95% CI 2.47–171.07; *p* = 0.005; Table S9). Importantly, only one death was reported in the reference group, skewing the results.

In the TriNetX cohort, there were 273, 1110, and 99 patients in each of the AC-only, AP-only, and dual groups, respectively, after matching. Reoperation HRs were 1.56 for AC only (95% CI 0.90–2.71; *p* = 0.107) and 1.51 for AP only (95% CI 1.10–2.06; *p* = 0.010). Because of small event counts, the dual-therapy reoperation HR could not be estimated. AP-only exposure was also associated with higher 30-day mortality in TriNetX (HR 1.58, 95% CI 1.22–2.06; *p* < 0.001). No class-specific thrombotic comparison was statistically significant (Table S9).

For the competing-risk analyses, at 90 days, the unmatched TriNetX reoperation cumulative incidence function (CIF) was 9.75% with any AC/AP and 7.16% without (Figure S2A). These estimates were consistent with the primary comparison but did not test the stability of the matched HR of 1.52 in the primary analysis. Single-center CIFs were 12.62% and 13.11%, respectively. The unadjusted Fine–Gray subdistribution hazard ratio (sHR) was 0.95 (95% CI 0.40–2.27; *p* = 0.911), with Gray’s test *p* = 0.912 (Figure S2B). In the single-center cohort at 90 days, there were 13 versus 8 reoperations and 17 versus 3 deaths before reoperation.

## 4. Discussion

In this retrospective study using both TriNetX and single-center cohorts, preoperative antithrombotic exposure was associated with increased 90-day repeat SDH surgery in the TriNetX propensity-score-matched cohort. However, this association was not reproduced in the single-center cohort. Within the single-center cohort, exposed patients had more baseline comorbidities, worse functional status at the time of discharge, and greater 30-day and overall mortality, although these associations attenuated after multivariable adjustment. Together, these findings suggest that preoperative antithrombotic exposure may identify a higher-risk SDH surgical population, but its association with postoperative outcomes varies by data source and endpoint ascertainment.

With TriNetX, a powered estimate was obtained, whereas the single-center cohort was smaller and likely reflected local surgical criteria and postoperative surveillance practices. In addition, TriNetX repeat surgery was based on procedural codes, whereas the institutional cohort used chart-reviewed events. These differences may explain why the reoperation association was not reproduced locally. The single-center cohort adds variables unavailable in large EHR databases, including presenting GCS, hematoma stage, maximum thickness, midline shift, and mRS, which assist with the contextualization of the TriNetX findings.

### 4.1. Mortality and Overall Postoperative Outcomes

The literature addressing the outcomes of patients with SDH and AC/AP exposure varies. In addition, findings vary by class and differing outcome definitions. In a propensity-matched chronic SDH cohort, Salih et al. found that long-term anticoagulation was not associated with longer hospital stays, periprocedural complications, or recurrence, supporting that anticoagulation alone may not consistently worsen perioperative outcomes [22]. Younsi et al. found comparable outcomes between patients who were exposed to antithrombotic therapy and those who were not and instead found comorbidity burden as an important factor [20]. Similarly, Fornebo et al., Poon et al., and Kerttula et al. found that antithrombotic exposure was not clearly associated with worse mortality or recurrence outcomes in chronic SDH cohorts [21,23,24]. Overall, comorbidity burden and baseline clinical status may confound the relationship between antithrombotic exposure and mortality after SDH evacuation.

Although 30-day mortality was not significantly different in the TriNetX cohort, the single-center cohort showed higher early mortality among exposed patients in univariable Cox analysis. This association was attenuated in multivariable analysis, consistent with the interpretation that early mortality risk may be driven by age and comorbidity rather than independent AC/AP exposure. Likewise, overall survival differed in univariable analysis but was no longer associated with antithrombotic exposure after adjustment. Because the adjusted 30-day mortality model included only 19 deaths, this analysis was vulnerable to overfitting and should be interpreted as exploratory.

### 4.2. Postoperative Thrombotic Complications

Our study found that AC/AP exposure was not significantly associated with 14-day thrombotic outcomes. This finding should be interpreted cautiously, as patients receiving AC/AP therapy often have multiple comorbidities that independently increase risk. Prior chronic SDH studies have reported an association with increased thromboembolic risk after SDH evacuation and antithrombotic interruption, and others have suggested that early antithrombotic resumption may prevent higher thromboembolic frequency without an increased recurrence risk [21,23,25,26]. However, after propensity-score matching, we observed no statistically significant difference in early postoperative thrombotic outcomes. Matching likely reduced confounding from conditions that serve as the initial indication for antithrombotic therapy. The 14-day window may also be too short to detect later events. The single-center thrombotic event rate was also numerically higher among exposed patients, but event counts were sparse.

### 4.3. Antithrombotic Exposure and Reoperation Risk

In the TriNetX cohort, the primary finding was that preoperative AC/AP exposure was associated with higher 90-day repeat SDH surgical intervention. This association was not reproduced in the single-center cohort, emphasizing that reoperation risk may depend on case mix or local operative practice. Several chronic SDH studies have reported similar associations. Choi et al. reported that preoperative antithrombotic medication was independently associated with chronic SDH recurrence after burr-hole surgery [27]. Motiei-Langroudi et al. studied variables associated with repeat surgery following chronic SDH evacuation and identified clopidogrel or warfarin use as the strongest predictors of reoperation, along with hematoma loculation and the extent of evacuation [16]. Because aspirin was included in the pooled AC/AP exposure, aspirin-specific evidence is particularly relevant; recent randomized data suggest that perioperative aspirin discontinuation may not reduce recurrence after surgically treated chronic SDH [28]. Wada et al. reported that antiplatelet therapy significantly influenced postoperative recurrence in a multicenter cohort [29].

Numerous mechanisms have been proposed for SDH recurrence. Additional nuances are observed based upon the stage of SDH. Some include prolonged microvascular bleeding, impaired clot formation, delayed healing, and residual expansion; however, in TriNetX, we were not able to assess reversal, hematoma features, or radiographic recurrence. Prior studies have identified numerous predictors of recurrence encompassing radiographic and operative factors, such as location, thickness, and midline shift, suggesting that SDH recurrence is multifactorial [16,17,30]. Repeat surgery is clinically meaningful, as Forster et al. found that reoperation after chronic SDH surgery was associated with worse postoperative quality of life [31]. These studies, along with our study, highlight the importance of identifying risk factors for repeat SDH evacuation.

The class-specific sensitivity analysis did not establish whether the pooled reoperation association was driven by a single class. AC-only and AP-only reoperation estimates were similar, while dual-therapy events were sparse. This analysis is limited by using newly matched TriNetX populations with small sample sizes and sparse events, as well as residual imbalance after matching that may differ from the primary cohorts.

### 4.4. Comparison with Prior Chronic SDH Literature

Not all studies have identified antithrombotic therapy as an independent predictor of recurrence. Several studies found no independent association between antithrombotic exposure and recurrence or reoperation and instead point towards the importance of comorbidities [32,33,34]. Larger studies also remain heterogeneous, with meta-analyses showing associations between antithrombotic exposure and recurrence or thrombotic events, while data derived from trials have suggested that antithrombotic exposure does not increase recurrence after burr-hole evacuation [35,36,37,38]. These findings could be the result of differences in study design, definitions for recurrence, or patient selection criteria. Medication management, such as the timing of postoperative resumption, likely plays a critical role. Additionally, some studies used radiographic recurrence in chronic SDH, whereas we evaluated repeat operation. As such, our results reflect clinically significant recurrence requiring another surgery.

### 4.5. Clinical Implications and Perioperative Antithrombotic Management

Operative SDH is increasingly encountered in older patients on chronic antithrombotic therapy. Exposed patients may need closer surveillance, but the decision to interrupt or resume antithrombotic agents should remain individualized to the patient. Importantly, in the primary pooled comparisons, the TriNetX cohort did not show significantly different mortality or thrombotic complications, while the single-center mortality associations attenuated after multivariable adjustment. Together, our findings indicate that management decisions should be weighed against the risk of repeat intervention and the need for protection from thrombosis [26,39,40,41].

### 4.6. Limitations

Several limitations should be considered. First, the retrospective design is susceptible to residual or unmeasured confounding, especially by indication for antithrombotic therapy, despite propensity-score matching. In addition, patient criteria for surgical selection in participating institutions reflect individual practices and may introduce selection bias. TriNetX study definitions rely on ICD, CPT, and RxNorm codes, rendering coding misclassification possible. Although patients were identified using nontraumatic SDH codes, unrecognized traumas may not have been excluded. Clinical and radiographic variables known to influence SDH recurrence, such as hematoma size, midline shift, laterality, and membrane/internal architecture, were not provided in TriNetX [17]. Postoperative residual hematoma or degree of evacuation was also unavailable, although these factors have been associated with reoperation [16]. Operative details such as drain placement were also unavailable, and combining twist-drill, burr-hole, and craniotomy procedures may have introduced additional heterogeneity [42]. This is important because drain placement after burr-hole evacuation has been shown to reduce recurrence and mortality in chronic SDH [18].

Additionally, the TriNetX cohort evaluated ICD-coded nontraumatic SDH, and the database is unable to distinguish between chronic, acute-on-chronic, and subacute SDH, limiting comparison to studies that were confined to chronic SDH. In addition, combining twist-drill, burr-hole, and craniotomy as modalities mixes different surgical cases and indications among the population. In our single-center analysis, craniotomy or craniectomy accounted for 72.8% of exposed and 68.9% of unexposed operations. Therefore, our findings should not be generalized to chronic SDH populations largely treated with burr-hole drainage. The single-center study is intended to provide additional context and not validate an identically defined multicenter population. Medication-related details, such as adherence, timing of last dose, reversal strategies, and timing of postoperative resumption, were not available. Additionally, over-the-counter aspirin use may have been incompletely captured. Antiplatelet users may have been misclassified as unexposed, and some patients receiving both classes may have been classified as AC only. Importantly, medication records do not establish adherence or ingestion. The resulting exposure misclassification may bias the estimates or class-specific results, but its magnitude of impact cannot be determined in the present study. In this study, our outcome was repeat surgical intervention and not radiographic recurrence. Therefore, radiographic recurrences or hematomas managed nonoperatively were not captured. Lastly, thrombotic outcomes were identified using diagnostic codes, which may capture preexisting conditions. Potential variability in institutional coding, postoperative surveillance, follow-up periods, and possible care from organizations not contributory to TriNetX may lead to incomplete outcome capture. In addition, variation in site practices could not be accounted for.

The single-center cohort was limited by sample size, low event counts, retrospective chart abstraction, and residual confounding. The single-center cohort also differed from TriNetX in SDH selection and thrombotic event window, which limits comparisons across the cohorts. Studying the period of index hospitalization provides longer observation for those with prolonged stays but omits events after discharge, including those that occur within 14 days. By contrast, the TriNetX 14-day window may include events occurring after discharge but excludes events after postoperative day 14, including during prolonged hospitalization. Differences in these windows therefore introduce observational bias, and absolute outcome rates should not be interpreted as equivalent among the two cohorts. Although mortality was studied as a competing risk, the TriNetX CIFs were unmatched and cannot establish the stability of the HR presented in the primary analysis. Single-center Fine–Gray values were unadjusted, and the conclusions are limited by the low event counts.

## 5. Conclusions

In a multicenter cohort of patients undergoing surgical evacuation of SDH, preoperative exposure to anticoagulants or antiplatelets was significantly associated with repeat surgery within 90 days, but there was no association with 30-day mortality or early thrombotic events. In the single-center cohort, the reoperation association was not reproduced. Patients exposed to preoperative antithrombotic therapy showed worse discharge functional status and higher unadjusted early and overall mortality, but these associations attenuated after multivariable adjustment. Our findings demonstrate that preoperative antithrombotic exposure may identify a complex, high-risk SDH population and support individualized postoperative surveillance and antithrombotic management.

## Figures and Tables

**Table 1:** Baseline characteristics before and after matching.

Variable	Before Matching AC/AP (*n* = 1456)	Before Matching Control (*n* = 14,195)	SMD ^a^	After Matching AC/AP (*n* = 1455)	After Matching Control (*n* = 1455)	SMD ^a^
**Demographics**						
Age, years	69.0 (12.1)	64.7 (14.5)	0.317	69.0 (12.1)	70.6 (10.9)	0.14
Male	1026 (70.5)	9948 (70.2)	0.007	1026 (70.5)	1031 (70.9)	0.008
Female	430 (29.5)	4230 (29.8)	0.007	429 (29.5)	424 (29.1)	0.008
Not Hispanic or Latino	1214 (83.4)	10,920 (77.0)	0.16	1214 (83.4)	1235 (84.9)	0.04
Unknown Ethnicity	149 (10.2)	2240 (15.8)	0.166	149 (10.2)	147 (10.1)	0.005
Hispanic or Latino	93 (6.4)	1019 (7.2)	0.032	92 (6.3)	73 (5.0)	0.056
White	1008 (69.2)	9990 (70.5)	0.027	1008 (69.3)	1025 (70.4)	0.025
Unknown Race	71 (4.9)	726 (5.1)	0.011	71 (4.9)	65 (4.5)	0.02
Black or African American	221 (15.2)	1967 (13.9)	0.037	221 (15.2)	196 (13.5)	0.049
Other Race	57 (3.9)	497 (3.5)	0.022	56 (3.8)	47 (3.2)	0.033
Asian	90 (6.2)	836 (5.9)	0.012	90 (6.2)	109 (7.5)	0.052
Native Hawaiian or Other Pacific Islander	10 (0.7)	105 (0.7)	0.006	10 (0.7)	10 (0.7)	<0.001
American Indian or Alaska Native	10 (0.7)	58 (0.4)	0.038	10 (0.7)	11 (0.8)	0.008
BMI ≥ 30 kg/m^2^	403 (27.7)	3498 (24.7)	0.068	402 (27.6)	398 (27.4)	0.006
**Comorbidities**						
Atrial fibrillation or flutter	392 (26.9)	1717 (12.1)	0.38	391 (26.9)	370 (25.4)	0.033
Other venous embolism and thrombosis	202 (13.9)	829 (5.8)	0.272	201 (13.8)	169 (11.6)	0.066
Ischemic heart diseases	665 (45.7)	3060 (21.6)	0.527	664 (45.6)	672 (46.2)	0.011
Peripheral vascular disease	128 (8.8)	677 (4.8)	0.16	128 (8.8)	119 (8.2)	0.022
Cerebral infarction	289 (19.8)	1320 (9.3)	0.302	288 (19.8)	288 (19.8)	<0.001
Transient ischemic attack	98 (6.7)	543 (3.8)	0.13	98 (6.7)	81 (5.6)	0.049
Pulmonary embolism	72 (4.9)	308 (2.2)	0.15	72 (4.9)	63 (4.3)	0.029
Essential hypertension	1036 (71.2)	7861 (55.4)	0.33	1035 (71.1)	1056 (72.6)	0.032
Type 2 diabetes mellitus	524 (36.0)	3365 (23.7)	0.27	523 (35.9)	532 (36.6)	0.013
Chronic kidney disease	307 (21.1)	1848 (13.0)	0.215	306 (21.0)	289 (19.9)	0.029
Heart failure	366 (25.1)	1461 (10.3)	0.396	365 (25.1)	343 (23.6)	0.035
Liver disease	179 (12.3)	1488 (10.5)	0.057	178 (12.2)	168 (11.5)	0.021
Alcohol-related disorders	146 (10.0)	1790 (12.6)	0.082	146 (10.0)	131 (9.0)	0.035
Nicotine dependence	227 (15.6)	2233 (15.7)	0.004	227 (15.6)	214 (14.7)	0.025
Mechanical heart valves	69 (4.7)	260 (1.8)	0.164	68 (4.7)	55 (3.8)	0.044

Categorical variables are presented as *n* (%). Age is presented as mean (SD). ^a^ SMD, standardized mean difference; values < 0.10 were the prespecified benchmark for covariate balance. Abbreviations: AC/AP, anticoagulant/antiplatelet; BMI, body mass index; SD, standard deviation; SMD, standardized mean difference.

**Table 2:** Single-center clinical, radiographic, laboratory, operative, and functional characteristics.

Variable	No AC/AP (*n* = 61)	Any AC/AP (*n* = 103)	*p* Value
Patient factors			
Age, years	69 [58–76]	78 [68–82]	**<0.001 ^†^**
Male sex	40 (65.6%)	78 (75.7%)	0.162
BMI, kg/m^2^	26.1 [23.6–29.4]	26.8 [24.9–31]	**0.035 ^†^**
Smoking status: Never	31 (50.8%)	44 (42.7%)	**0.001**
Smoking status: Former	18 (29.5%)	54 (52.4%)	
Smoking status: Current	12 (19.7%)	5 (4.9%)	
Hypertension	37 (60.7%)	83 (80.6%)	**0.005**
Atrial fibrillation	2 (3.3%)	29 (28.2%)	**<0.001**
Coronary artery disease	3 (4.9%)	51 (49.5%)	**<0.001**
Chronic kidney disease	6 (9.8%)	12 (11.7%)	0.719
Prior stroke/TIA	4 (6.6%)	16 (15.5%)	0.090
Alcoholism	10 (16.4%)	1 (1.0%)	**<0.001 ^‡^**
Presentation and laboratory values			
Preoperative GCS	15 [15–15]	15 [15–15] (*n* = 99)	0.927 ^†^
INR at presentation	1 [1–1.1] (*n* = 52)	1.1 [1–1.4] (*n* = 88)	**<0.001 ^†^**
Platelet count at presentation	242 [196–277] (*n* = 57)	208 [156–259] (*n* = 98)	**0.036 ^†^**
Radiographic findings			
SDH chronicity/type: Acute	13 (21.3%)	17 (16.5%)	0.050
SDH chronicity/type: Subacute	19 (31.1%)	16 (15.5%)	-
SDH chronicity/type: Chronic	6 (9.8%)	11 (10.7%)	-
SDH chronicity/type: Mixed	23 (37.7%)	59 (57.3%)	-
SDH laterality: Left	19 (31.1%)	42 (40.8%)	0.141
SDH laterality: Right	25 (41.0%)	27 (26.2%)	-
SDH laterality: Bilateral	17 (27.9%)	34 (33.0%)	-
Maximum hematoma thickness, mm	20 [16–25] (*n* = 53)	19.5 [15–25] (*n* = 88)	0.343 ^†^
Midline shift, mm	8 [4.8–12] (*n* = 51)	8 [4–11.5] (*n* = 83)	0.747 ^†^
Operative features			
Operative approach: Burr hole	16 (26.2%)	25 (24.3%)	0.746
Operative approach: Craniotomy/craniectomy	42 (68.9%)	75 (72.8%)	-
Operative approach: SEPS/twist-drill-type drainage	3 (4.9%)	3 (2.9%)	-
Functional status			
Discharge mRS	3 [2–4]	4 [3,4]	**0.002 ^†^**
Poor discharge mRS (4–6)	22 (36.1%)	62 (60.2%)	**0.003**

Bold values indicate *p* < 0.05. Values are presented as *n* (%) or median [IQR], unless otherwise indicated. Available *n* is shown where observations are missing. *p* values were calculated using Pearson chi-square tests unless indicated by ^†^ (Wilcoxon rank-sum test) or ^‡^ (Fisher exact test). Abbreviations: AC/AP, anticoagulant/antiplatelet; BMI, body mass index; GCS, Glasgow Coma Scale; INR, international normalized ratio; IQR, interquartile range; mRS, modified Rankin Scale; SDH, subdural hematoma; SEPS, subdural evacuating port system; TIA, transient ischemic attack.

**Table 3:** Cross-cohort postoperative outcome summary.

Panel A. TriNetX cohort				
**Outcome**	**Any AC/AP** **Events/*N* (%)**	**No AC/AP** **Events/*N* (%)**	**Effect Estimate** **(95% CI)**	***p *** **Value**
90-day reoperation	141/1455 (9.7)	94/1455 (6.5)	HR 1.52 (1.17–1.98)	0.001
30-day mortality	176/1455 (12.1)	157/1455 (10.8)	HR 1.13 (0.91–1.40)	0.281
14-day thrombotic events	174/1455 (12.0)	149/1455 (10.2)	RR 1.17 (0.95–1.44)	0.140
Panel B. Single-center cohort				
**Outcome**	**Any AC/AP** **Events/*N* (%)**	**No AC/AP** **Events/*N* (%)**	**Effect Estimate** **(95% CI)**	***p *** **Value**
90-day reoperation	13/103 (12.6)	8/61 (13.1)	HR 1.01 (0.42–2.44)	0.983
30-day mortality	18/103 (17.5)	1/61 (1.6)	aHR 6.09 (0.73–51.12)	0.096
In-hospital thrombotic events	9/103 (8.7)	2/61 (3.3)	RR 2.67 (0.60–11.93)	0.214

Values are presented as Events/*N* (%). Abbreviations: AC/AP, anticoagulant/antiplatelet; aHR, adjusted hazard ratio; CI, confidence interval; HR, hazard ratio; RR, risk ratio.

## Data Availability

TriNetX data are available through the TriNetX platform, subject to institutional access and data-use requirements, and cannot be publicly redistributed by the authors. Deidentified data from the single-center cohort may be available from the corresponding author upon reasonable request. The data are not publicly available due to ethical and data-use restrictions placed on protected patient data and the TriNetX platform.

## Supplementary Materials

The following supporting information can be downloaded at https://www.mdpi.com/article/10.3390/brainsci16101084/s1, Figure S1: Overall survival in the single-center cohort stratified by anticoagulant or antiplatelet exposure; Table S1: TriNetX encounter, diagnostic, and prescription codes used to define cohorts; Table S2: Presenting symptoms by anticoagulant or antiplatelet exposure; Table S3: Thrombotic event subtypes in both cohorts; Table S4: Univariable Cox proportional hazards analysis of 30-day postoperative mortality in the single-center cohort; Table S5: Multivariable Cox proportional hazards analysis of 30-day postoperative mortality in the single-center cohort; Table S6: Univariable Cox proportional hazards analysis of overall survival in the single-center cohort; Table S7: Multivariable Cox proportional hazards analysis of overall survival in the single-center cohort; Table S8: Single-center characteristics by antithrombotic class; Table S9: Drug-class sensitivity analyses in both cohorts; Figure S2: Competing-risk analysis of 90-day repeat SDH surgery.

## Abbreviations

The following abbreviations are used in this manuscript:

AC/AP	Anticoagulant/antiplatelet
BMI	Body mass index
CAD	Coronary artery disease
CI	Confidence interval
CKD	Chronic kidney disease
CPT	Current Procedural Terminology
EHR	Electronic health record
GCS	Glasgow Coma Scale
HR	Hazard ratio
ICD-10	International Classification of Diseases, Tenth Revision
INR	International normalized ratio
IQR	Interquartile range
mRS	Modified Rankin Scale
RR	Risk ratio
SD	Standard deviation
SDH	Subdural hematoma
SEPS	Subdural evacuating port system
SMD	Standardized mean difference
STROBE	Strengthening the Reporting of Observational Studies in Epidemiology
TIA	Transient ischemic attack
