# Supplementary material for: Preoperative Antithrombotic Exposure and Postoperative Outcomes Following Nontraumatic Subdural Hematoma Evacuation: A Multicenter Electronic Health Record Analysis with Single-Center Context
Source: Brain Sci. 2026 Oct 9;16(10):1084. doi: 10.3390/brainsci16101084 (PMC13702731; doi:10.3390/brainsci16101084)
Supplement: Supplementary file 1 [file brainsci-16-01084-s001.zip › brainsci-4549351-supplementary.pdf]

Table S1: TriNetX encounter, diagnostic, and prescription codes used to define cohorts

Cohort	Must Have	Cannot Have
**AC/AP-exposed operative nontraumatic SDH cohort**	Age ≥ 18 years<br>AND<br>UMLS:CPT:61108 (twist drill hole(s) for subdural, intracerebral, or ventricular puncture; for evacuation and/or drainage of subdural hematoma) OR UMLS:CPT:61154 (burr hole(s) with evacuation and/or drainage of hematoma, extradural or subdural) OR UMLS:CPT:61312 (craniectomy or craniotomy for evacuation of hematoma, supratentorial; extradural or subdural) OR UMLS:CPT:61314 (craniectomy or craniotomy for evacuation of hematoma, infratentorial; extradural or subdural) between September 1, 2005 and September 1, 2025:<br>**AND**<br>UMLS:ICD10CM:I62.0 (nontraumatic subdural hemorrhage) within 1 week of operative management<br>AND<br>at least one within one week before operation: UMLS:RxNorm:11289 (warfarin [Oral Product]) OR UMLS:RxNorm:1364430 (apixaban [Oral Product]) OR UMLS:RxNorm:10594 (ticlopidine [Oral Product]) OR UMLS:RxNorm:1114195 (rivaroxaban [Oral Product]) OR UMLS:RxNorm:1037042 (dabigatran etexilate [Oral Product]) OR UMLS:RxNorm:1599538 (edoxaban [Oral Product]) OR UMLS:RxNorm:1191 (aspirin [Oral Product]) OR UMLS:RxNorm:32968 (clopidogrel [Oral Product]) OR UMLS:RxNorm:613391 (prasugrel [Oral Product]) OR UMLS:RxNorm:1116632 (ticagrelor [Oral Product]).	UMLS:RxNorm:10106 (streptokinase) OR UMLS:RxNorm:11055 (urokinase) OR UMLS:RxNorm:259280 (tenecteplase) OR UMLS:RxNorm:352374 (drotrecogin alfa) OR UMLS:RxNorm:40028 (anistreplase) OR UMLS:RxNorm:76895 (reteplase) OR UMLS:RxNorm:772 (ancrod) OR UMLS:RxNorm:8834 (protein C) OR UMLS:RxNorm:8410 (alteplase).
**Unexposed operative nontraumatic SDH cohort**	Age >= 18 years<br>AND<br>UMLS:CPT:61108 (twist drill hole(s) for subdural, intracerebral, or ventricular puncture; for evacuation and/or drainage of subdural hematoma) OR UMLS:CPT:61154 (burr hole(s) with evacuation and/or drainage of hematoma, extradural or subdural) OR UMLS:CPT:61312 (craniectomy or craniotomy for evacuation of hematoma, supratentorial; extradural or subdural) OR UMLS:CPT:61314 (craniectomy or	UMLS:RxNorm:321208 (fondaparinux) OR UMLS:RxNorm:2110605 (caplacizumab) OR UMLS:RxNorm:1311089 (defibrotide) OR UMLS:RxNorm:1599538 (edoxaban) OR UMLS:RxNorm:1364430 (apixaban) OR UMLS:RxNorm:1114195 (rivaroxaban) OR UMLS:RxNorm:60819 (bivalirudin) OR UMLS:RxNorm:237057 (lepirudin) OR UMLS:RxNorm:15202 (argatroban) OR UMLS:RxNorm:114934 (desirudin) OR UMLS:RxNorm:1037042 (dabigatran

Cohort	Must Have	Cannot Have
	craniotomy for evacuation of hematoma, infratentorial; extradural or subdural) between September 1, 2005 and September 1, 2025 AND UMLS:ICD10CM:I62.0 (nontraumatic subdural hemorrhage) within 1 week of operative management	etexilate) OR UMLS:RxNorm:8834 (protein C) OR UMLS:RxNorm:8410 (alteplase) OR UMLS:RxNorm:772 (ancrod) OR UMLS:RxNorm:76895 (reteplase) OR UMLS:RxNorm:40028 (anistreplase) OR UMLS:RxNorm:352374 (drotrecogin alfa) OR UMLS:RxNorm:259280 (tenecteplase) OR UMLS:RxNorm:11055 (urokinase) OR UMLS:RxNorm:10106 (streptokinase) OR UMLS:RxNorm:83929 (abciximab) OR UMLS:RxNorm:8814 (epoprostenol) OR UMLS:RxNorm:75635 (eptifibatide) OR UMLS:RxNorm:73137 (tirofiban) OR UMLS:RxNorm:613391 (prasugrel) OR UMLS:RxNorm:40138 (iloprost) OR UMLS:RxNorm:3521 (dipyridamole) OR UMLS:RxNorm:1191 (aspirin) OR UMLS:RxNorm:1537034 (vorapaxar) OR UMLS:RxNorm:1656052 (cangrelor) OR UMLS:RxNorm:1729002 (selexipag) OR UMLS:RxNorm:21107 (cilostazol) OR UMLS:RxNorm:32968 (clopidogrel) OR UMLS:RxNorm:343048 (treprostinil) OR UMLS:RxNorm:11289 (warfarin) OR UMLS:RxNorm:154 (acenocoumarol) OR UMLS:RxNorm:1598 (dicumarol) OR UMLS:RxNorm:1009 (antithrombin III) OR UMLS:RxNorm:5224 (heparin) OR UMLS:RxNorm:67108 (enoxaparin) OR UMLS:RxNorm:67109 (dalteparin) OR UMLS:RxNorm:69646 (tinzaparin) OR UMLS:RxNorm:78484 (danaparoid) OR UMLS:RxNorm:10594 (ticlopidine) OR UMLS:RxNorm:1116632 (ticagrelor).

Abbreviations: *AC/AP*, anticoagulant/antiplatelet; *CPT*, Current Procedural Terminology; *ICD-10-CM*, International Classification of Diseases, Tenth Revision, Clinical Modification; *SDH*, subdural hematoma.

Table S2: Presenting symptoms by anticoagulant or antiplatelet exposure

Variable	No AC/AP exposure	Any AC/AP exposure	P value
Altered mental status	16 (26.2%)	31 (30.1%)	0.597
Aphasia	3 (4.9%)	6 (5.8%)	1.000†
Balance or walking problems	13 (21.3%)	20 (19.4%)	0.770
Confusion	9 (14.8%)	16 (15.5%)	0.893
Dizziness	4 (6.6%)	12 (11.7%)	0.288
Headache	28 (45.9%)	55 (53.4%)	0.353
Incontinence	1 (1.6%)	2 (1.9%)	1.000†
Memory dysfunction	1 (1.6%)	0 (0.0%)	0.372†
Nausea or vomiting	5 (8.2%)	9 (8.7%)	0.905
Losing consciousness	7 (11.5%)	4 (3.9%)	0.102†
Seizures	3 (4.9%)	1 (1.0%)	0.146†
Sleepiness	1 (1.6%)	2 (1.9%)	1.000†
Weakness	19 (31.1%)	37 (35.9%)	0.533
Sensory changes	1 (1.6%)	3 (2.9%)	1.000†
Vision change	2 (3.3%)	4 (3.9%)	1.000†

Values are presented as n (%).

P values were calculated using chi-square tests unless marked with †, indicating Fisher exact test.

Abbreviations: AC/AP = anticoagulant/antiplatelet.

Table S3: Thrombotic event subtypes in both cohorts

Outcome	TriNetX AC/AP vs no AC/AP	Single-center AC/AP vs no AC/AP
Ischemic stroke	99/1,455 (6.8%) vs 84/1,455 (5.8%)	3 (2.9%) vs 1 (1.6%)
Myocardial infarction	33/1,455 (2.3%) vs 29/1,455 (2.0%)	1 (1.0%) vs 0 (0.0%)
Deep venous thrombosis	44/1,455 (3.0%) vs 30/1,455 (2.1%)	4 (3.9%) vs 1 (1.6%)
Pulmonary embolism	26/1,455 (1.8%) vs 25/1,455 (1.7%)	1 (1.0%) vs 0 (0.0%)
Composite thrombotic event	174/1,455 (12.0%) vs 149/1,455 (10.2%)	9 (8.7%) vs 2 (3.3%)

Values are presented as n (%).

Table S4: Univariable Cox proportional hazards analysis of 30-day postoperative mortality in the single-center cohort

Predictor/covariate	N included in model	Events/N	HR	95% CI lower limit	95% CI upper limit	P value
AC/AP Exposure: Yes vs No	164	19/164	11.479	1.532	86.001	**0.0175**
Age	164	19/164	1.077	1.024	1.133	**0.0042**
BMI	164	19/164	1.040	0.975	1.109	0.2353
Tobacco: Current vs Never	164	19/164	0.430	0.055	3.359	0.4209
Tobacco: Former vs Never	164	19/164	0.814	0.321	2.062	0.6642
HTN	164	19/164	3.245	0.750	14.046	0.1153
Afib	164	19/164	2.072	0.787	5.452	0.1401
CAD	164	19/164	2.986	1.201	7.425	0.0186
INR at presentation	140	17/140	1.104	0.849	1.435	0.4599
Type: Mixed vs Acute	164	19/164	0.394	0.143	1.087	0.0721
Type: Subacute vs Acute	164	19/164	0.481	0.141	1.644	0.2434

Bold values indicate $p < 0.05$.

Abbreviations: AC/AP, anticoagulant/antiplatelet; BMI, body mass index; HTN, hypertension; Afib, atrial fibrillation; CAD, coronary artery disease; INR, International Normalized Ratio.

Table S5: Multivariable Cox proportional hazards analysis of 30-day postoperative mortality in the single-center cohort

Predictor/covariate	N included in model	Events/N	HR	95% CI lower limit	95% CI upper limit	P value
AC/AP Exposure: Yes vs No	164	19/164	6.091	0.726	51.117	0.0960
Age	164	19/164	1.043	0.986	1.103	0.1396
Tobacco: Current vs Never	164	19/164	1.079	0.127	9.131	0.9444
Tobacco: Former vs Never	164	19/164	0.574	0.198	1.665	0.3068
HTN	164	19/164	2.361	0.524	10.642	0.2636
Afib	164	19/164	1.340	0.457	3.933	0.5940
CAD	164	19/164	1.609	0.597	4.338	0.3470

Bold values indicate $p < 0.05$.
Abbreviations: AC/AP, anticoagulant/antiplatelet; HTN, hypertension; Afib, atrial fibrillation; CAD, coronary artery disease.

Table S6: Univariable Cox proportional hazards analysis of overall survival in the single-center cohort

Predictor/covariate	N included in model	Events/N	HR	95% CI lower limit	95% CI upper limit	P value
AC/AP Exposure: Yes vs No	164	71/164	2.256	1.328	3.832	0.0026
Age	164	71/164	1.083	1.054	1.113	<.0001
BMI	164	71/164	1.012	0.966	1.060	0.6152
Tobacco: Current vs Never	164	71/164	0.405	0.155	1.058	0.0650
Tobacco: Former vs Never	164	71/164	1.207	0.740	1.968	0.4509
HTN	164	71/164	2.381	1.307	4.336	0.0046
Afib	164	71/164	1.845	1.047	3.250	0.0341
CAD	164	71/164	1.786	1.100	2.901	0.0190
INR at presentation	140	63/140	1.085	0.906	1.300	0.3746
Type: Chronic vs Acute	164	71/164	0.692	0.247	1.934	0.4822
Type: Mixed vs Acute	164	71/164	0.984	0.528	1.837	0.9604
Type: Subacute vs Acute	164	71/164	1.091	0.530	2.246	0.8130
Binary mRS: 4 to 6 vs 0 to 3	164	71/164	4.008	2.386	6.731	<.0001

Bold values indicate p < 0.05.
Abbreviations: AC/AP, anticoagulant/antiplatelet; BMI, body mass index; HTN, hypertension; Afib, atrial fibrillation; CAD, coronary artery disease; INR, International Normalized Ratio; mRS, modified Rankin scale.

Table S7: Multivariable Cox proportional hazards analysis of overall survival in the single-center cohort

Predictor/covariate	N included in model	Events/N	HR	95% CI lower limit	95% CI upper limit	P value
AC/AP Exposure: Yes vs No	164	71/164	1.185	0.605	2.324	0.6204
Age	164	71/164	1.061	1.029	1.095	0.0002
Tobacco: Current vs Never	164	71/164	0.783	0.252	2.440	0.6736
Tobacco: Former vs Never	164	71/164	1.203	0.697	2.078	0.5063
HTN	164	71/164	1.754	0.917	3.355	0.0893
Afib	164	71/164	1.113	0.601	2.061	0.7338
CAD	164	71/164	1.128	0.637	1.997	0.6797
Binary mRS: 4 to 6 vs 0 to 3	164	71/164	3.343	1.929	5.793	<.0001

Bold values indicate $p < 0.05$.
Abbreviations: AC/AP, anticoagulant/antiplatelet; HTN, hypertension; Afib, atrial fibrillation; CAD, coronary artery disease; mRS, modified Rankin scale.

Table S8. Single-center characteristics by antithrombotic class

Variable	No AC/AP (n=61)	AC only (n=20)	AP only (n=63)	Dual AC+AP (n=20)	Global p
**Patient factors**					
Age, years	69.0 [58.0, 76.0]	71.5 [64.0, 81.5]	78.0 [68.0, 81.5]	79.5 [74.0, 82.2]	<0.001†
Sex: Female	21/61 (34.4%)	4/20 (20.0%)	16/63 (25.4%)	5/20 (25.0%)	0.535
Sex: Male	40/61 (65.6%)	16/20 (80.0%)	47/63 (74.6%)	15/20 (75.0%)	
BMI, kg/m²	26.1 [23.6, 29.4]	28.7 [25.8, 35.7]	26.7 [24.9, 30.8]	26.2 [24.7, 28.7]	0.034†
Smoking status: Never	31/61 (50.8%)	8/20 (40.0%)	29/63 (46.0%)	7/20 (35.0%)	0.025‡
Smoking status: Former	18/61 (29.5%)	12/20 (60.0%)	30/63 (47.6%)	12/20 (60.0%)	
Smoking status: Current	12/61 (19.7%)	0/20 (0.0%)	4/63 (6.3%)	1/20 (5.0%)	
Hypertension	37/61 (60.7%)	17/20 (85.0%)	50/63 (79.4%)	16/20 (80.0%)	0.046
Diabetes	14/61 (23.0%)	7/20 (35.0%)	16/63 (25.4%)	9/20 (45.0%)	0.228
Atrial fibrillation	2/61 (3.3%)	11/20 (55.0%)	8/63 (12.7%)	10/20 (50.0%)	<0.001‡
Coronary artery disease	3/61 (4.9%)	6/20 (30.0%)	31/63 (49.2%)	14/20 (70.0%)	<0.001
Kidney disease	6/61 (9.8%)	3/20 (15.0%)	7/63 (11.1%)	2/20 (10.0%)	0.946‡
Prior stroke or TIA	4/61 (6.6%)	3/20 (15.0%)	10/63 (15.9%)	3/20 (15.0%)	0.360‡
Alcoholism	10/61 (16.4%)	1/20 (5.0%)	0/63 (0.0%)	0/20 (0.0%)	<0.001‡
Liver disease	0/61 (0.0%)	0/20 (0.0%)	1/63 (1.6%)	0/20 (0.0%)	1.000‡
Cancer	6/61 (9.8%)	2/20 (10.0%)	10/63 (15.9%)	3/20 (15.0%)	0.774‡
COPD	6/61 (9.8%)	3/20 (15.0%)	5/63 (7.9%)	2/20 (10.0%)	0.751‡
Peripheral arterial disease	0/61 (0.0%)	1/20 (5.0%)	0/63 (0.0%)	1/20 (5.0%)	0.058‡
Recorded comorbidity count	1.0 [1.0, 3.0]	3.0 [2.0, 4.0]	3.0 [2.0, 3.0]	4.0 [2.5, 4.2]	<0.001†
**Presentation and laboratory values**					
Preoperative GCS	15.0 [15.0, 15.0]	15.0 [15.0, 15.0] (n=19)	15.0 [15.0, 15.0] (n=61)	15.0 [14.0, 15.0] (n=19)	0.536†
INR at presentation	1.00 [1.00, 1.10] (n=52)	2.30 [1.42, 2.85] (n=18)	1.00 [1.00, 1.10] (n=53)	1.40 [1.10, 2.30] (n=17)	<0.001†
Platelet count at presentation	242.0 [196.0, 277.0] (n=57)	214.0 [153.0, 257.5] (n=19)	208.0 [160.0, 256.0] (n=61)	203.5 [162.8, 263.5] (n=18)	0.199†
**Radiographic findings**					
SDH chronicity: Acute	13/61 (21.3%)	2/20 (10.0%)	11/63 (17.5%)	4/20 (20.0%)	0.309‡
SDH chronicity: Subacute	19/61 (31.1%)	3/20 (15.0%)	10/63 (15.9%)	3/20 (15.0%)	
SDH chronicity: Chronic	6/61 (9.8%)	1/20 (5.0%)	9/63 (14.3%)	1/20 (5.0%)	
SDH chronicity: Mixed	23/61 (37.7%)	14/20 (70.0%)	33/63 (52.4%)	12/20 (60.0%)	
Laterality: Left	19/61 (31.1%)	8/20 (40.0%)	29/63 (46.0%)	5/20 (25.0%)	0.235
Laterality: Right	25/61 (41.0%)	4/20 (20.0%)	17/63 (27.0%)	6/20 (30.0%)	
Laterality: Bilateral	17/61 (27.9%)	8/20 (40.0%)	17/63 (27.0%)	9/20 (45.0%)	
Maximum hematoma thickness, mm	20.0 [16.0, 25.0] (n=53)	16.0 [15.0, 25.0] (n=17)	20.0 [15.2, 25.0] (n=54)	21.0 [17.0, 24.0] (n=17)	0.474†
Midline shift, mm	8.0 [4.8, 12.0] (n=51)	9.0 [4.5, 14.0] (n=17)	8.0 [4.5, 10.0] (n=51)	8.0 [3.0, 13.0] (n=15)	0.675†
**Operative approach**					

Variable	No AC/AP (n=61)	AC only (n=20)	AP only (n=63)	Dual AC+AP (n=20)	Global p
Burr hole	16/61 (26.2%)	3/20 (15.0%)	19/63 (30.2%)	3/20 (15.0%)	0.565‡
Craniotomy/craniectomy	42/61 (68.9%)	16/20 (80.0%)	43/63 (68.3%)	16/20 (80.0%)	
SEPS	3/61 (4.9%)	1/20 (5.0%)	1/63 (1.6%)	1/20 (5.0%)	

Bold values indicate $p < 0.05$.

Values are presented as median [IQR] or n/N (%), unless otherwise indicated. Available n is shown for variables with missing observations. P values were calculated using Pearson chi-square tests unless indicated by † (Kruskal–Wallis test) or ‡ (Fisher exact test). Abbreviations: AC, anticoagulant; AP, antiplatelet; BMI, body mass index; COPD, chronic obstructive pulmonary disease; GCS, Glasgow Coma Scale; INR, international normalized ratio; IQR, interquartile range; SDH, subdural hematoma; SEPS, subdural evacuating port system; TIA, transient ischemic attack.

Table S9. Drug-class sensitivity analyses in both cohorts

Panel A. TriNetX independently matched comparisons

Exposure	N exposed / none	90-day reoperation HR (95% CI)	30-day mortality HR (95% CI)	14-day thrombotic events RR (95% CI)
AC only	273 / 273	1.56 (0.90-2.71) p=0.107	0.93 (0.55-1.59) p=0.788	1.36 (0.93-1.98) p=0.109
AP only	1,110 / 1,110	1.51 (1.10-2.06) p=0.010	1.58 (1.22-2.06) p<0.001	1.14 (0.91-1.44) p=0.256
Dual AC+AP	99 / 99	Not reported*	1.40 (0.73-2.68) p=0.308	0.71 (0.36-1.40) p=0.315

*not reported because of small event counts

Panel B. Single-center unadjusted comparisons

Exposure	N exposed / none	90-day reoperation HR (95% CI)	30-day mortality HR (95% CI)	In-hospital thrombotic events RR (95% CI)
AC only	20 / 61	0.78 (0.17-3.68) p=0.755	9.56 (0.99-91.94) p=0.051	3.05 (0.46-20.27) p=0.254
AP only	63 / 61	1.00 (0.38-2.67) p=0.998	9.18 (1.16-72.47) p=0.035	2.42 (0.49-12.01) p=0.440
Dual AC+AP	20 / 61	1.30 (0.35-4.93) p=0.696	20.57 (2.47-171.07) p=0.005	3.05 (0.46-20.27) p=0.254

HRs are from unadjusted Cox models with Efron ties and Wald p values. RRs use log-Wald CIs and two-sided Fisher exact p values. Abbreviations: AC, anticoagulant; AP, antiplatelet; CI, confidence interval; DVT, deep venous thrombosis; HR, hazard ratio; PSM, propensity-score matching; RR, risk ratio; SMD, standardized mean difference; TIA, transient ischemic attack.

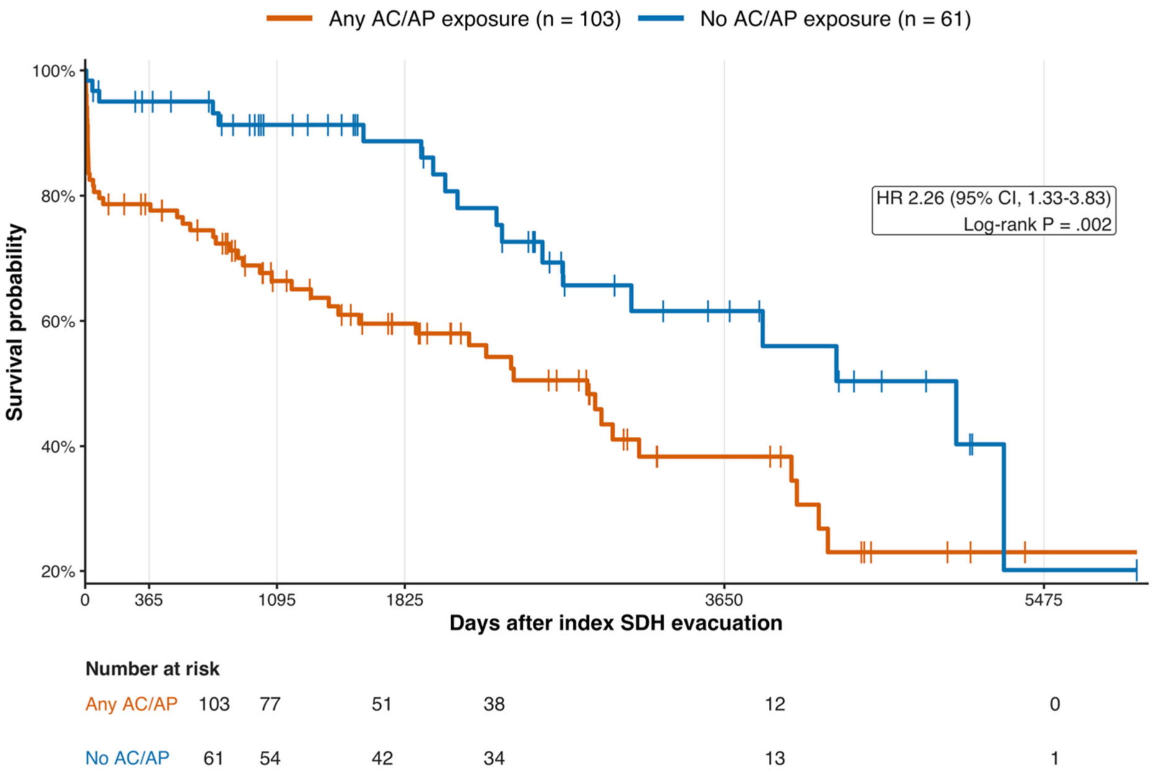

Figure S1: Overall survival in the single-center cohort stratified by anticoagulant or antiplatelet exposure. Kaplan-Meier curves comparing overall survival after index subdural hematoma evacuation among patients with and without preoperative antithrombotic exposure in the single-center cohort. Tick marks indicate censored observations. The hazard ratio reflects univariable Cox proportional hazards analysis, and the P value reflects the log-rank test. Abbreviations: AC/AP, anticoagulant/antiplatelet; CI, confidence interval; HR, hazard ratio; SDH, subdural hematoma.

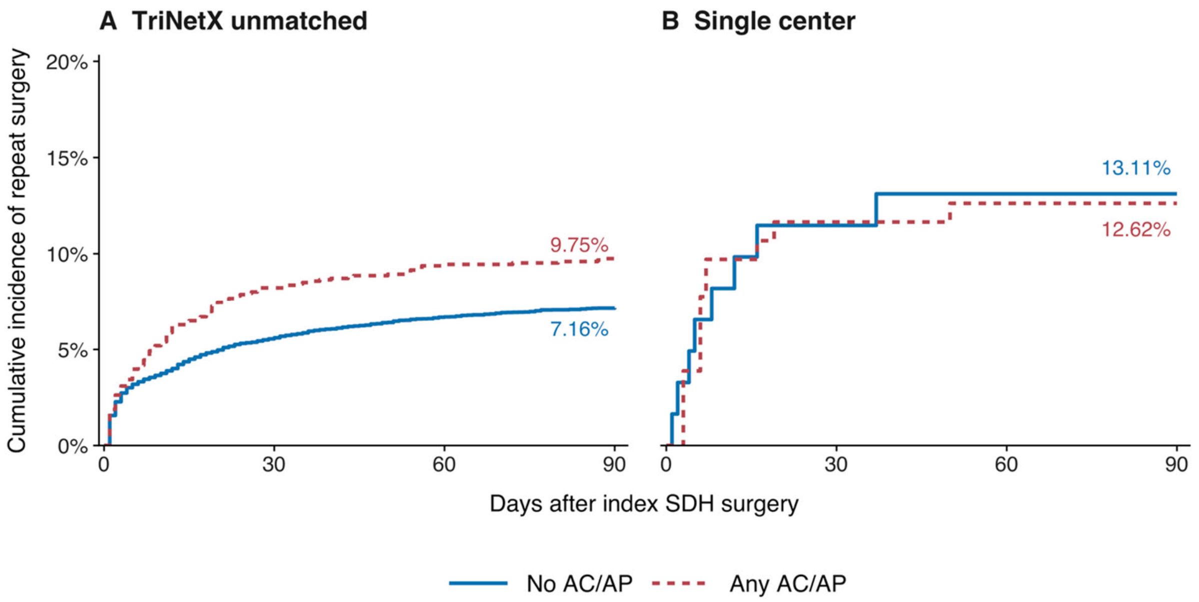

Figure S2. Competing-risk analysis of 90-day repeat SDH surgery. (A) TriNetX Aalen-Johansen cumulative-incidence functions from separate unmatched cohorts. (B) Institutional cumulative incidence among 103 patients with any AC/AP and 61 without. Abbreviations: AC/AP, anticoagulant/antiplatelet; CI, confidence interval; CIF, cumulative-incidence function; HR, hazard ratio; SDH, subdural hematoma; sHR, subdistribution hazard ratio.
